# Supplementary material for: Genome-Wide Association Study of Agronomic and Physiological Traits Related to Drought Tolerance in Potato
Source: Plants (Basel). 2023 Feb 7;12(4):734. doi: 10.3390/plants12040734 (PMC9963855; doi:10.3390/plants12040734)
Supplement: Supplementary file 1 [file plants-12-00734-s001.zip › Supplementary File 1.pdf]

Supplementary Table S1. Mean, standard deviation and phenotypic variance of 144 tetraploid potato varieties.

	2019			2020		
	Mean±SE	SD	σ^2	Mean±SE	SD	σ^2
SPAD50_C	45.13±0.349	4.18	17.49	42.00±0.347	4.16	17.34
NDVI50_C	0.88±0.001	0.01	0.0002	0.87±0.002	0.02	0.0004
SC50_C	424.07±15.427	185.13	34272.35	617.87±17.079	204.95	42003.42
FLUOR50_C	0.67±0.005	0.07	0.004	0.69±0.003	0.03	0.001
SPAD70_C	45.13±0.421	5.05	25.49	40.31±0.387	4.64	21.53
NDVI70_C	0.86±0.003	0.04	0.001	0.84±0.004	0.05	0.002
SC70_C	453.64±17.222	206.66	42708.85	501.71±14.696	176.35	31098.39
FLUOR70_C	0.69±0.004	0.05	0.002	0.72±0.003	0.04	0.001
Yield_C	5.56±0.169	2.03	4.10	4.51±0.146	1.75	3.05
TubNum_C	51.31±1.898	22.78	518.72	41.82±1.437	17.25	297.41
TubWeight_C	111.66±2.805	33.66	1133.08	114.30±2.988	35.86	1285.71
DryMatter_C	20.08±0.338	4.06	16.45	17.79±0.300	3.59	12.91
RS_C	0.20±0.013	0.16	0.02	0.16±0.010	0.12	0.01
Starch_C	13.11±0.347	4.17	17.37	10.76±0.308	3.69	13.64
Area_C	14.01±0.271	3.25	10.59	16.75±0.337	4.04	16.35
Perim_C	16.74±0.156	1.87	3.50	18.18±0.173	2.08	4.31
SPAD50_D	46.06±0.336	4.03	16.22	41.63±0.373	4.47	20.01
NDVI50_D	0.84±0.004	0.05	0.002	0.86±0.002	0.02	0.0006
SC50_D	283.44±12.079	144.94	21008.61	445.16±16.718	200.61	40246.29
FLUOR50_D	0.64±0.005	0.06	0.003	0.70±0.003	0.04	0.001
SPAD70_D	42.99±0.493	5.92	34.99	39.69±0.406	4.88	23.79
NDVI70_D	0.81±0.007	0.08	0.006	0.81±0.006	0.08	0.005
SC70_D	308.53±12.078	144.93	21005.9	362.38±17.309	207.71	43143.61
FLUOR70_D	0.62±0.005	0.06	0.003	0.68±0.003	0.04	0.001
Yield_D	2.13±0.079	0.95	0.904	2.66±0.098	1.18	1.38
TubNum_D	37.66±1.371	16.45	270.66	38.90±1.444	17.32	300.11
TubWeight_D	58.63±1.546	18.56	344.32	72.76±1.923	23.08	532.60
DryMatter_D	20.51±0.351	4.21	17.69	19.19±0.356	4.28	18.28
RS_D	0.16±0.007	0.09	0.007	0.16±0.008	0.10	0.009
Starch_D	13.55±0.360	4.32	18.68	12.20±0.366	4.39	19.3
Area_D	13.92±0.394	4.72	22.32	13.83±0.252	3.03	9.16
Perim_D	16.34±0.209	2.51	6.27	16.56±0.147	1.77	3.11
