# Supplementary material for: Genome-Wide Association Study of Agronomic and Physiological Traits Related to Drought Tolerance in Potato
Source: Plants (Basel). 2023 Feb 7;12(4):734. doi: 10.3390/plants12040734 (PMC9963855; doi:10.3390/plants12040734)
Supplement: Supplementary file 1 [file plants-12-00734-s001.zip › Supplementary File 2.pdf]

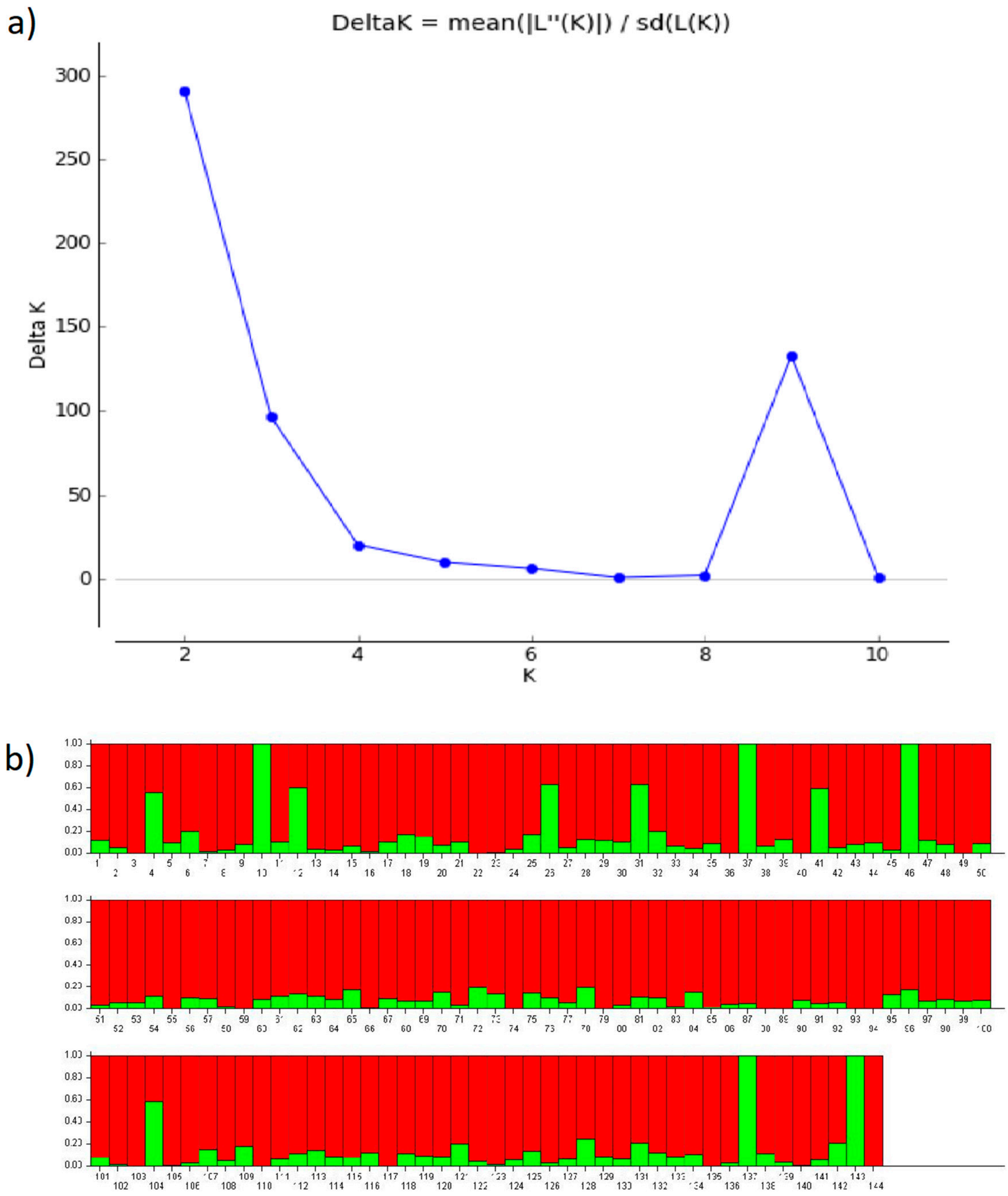

Supplementary Figure S1. (a) Delta K values over 10 runs and (b) bar plot displaying Q values obtained from STRUCTURE software in a population of 144 potato genotypes with a delta K=2.
