# Supplementary material for: Genome-Wide Association Study of Agronomic and Physiological Traits Related to Drought Tolerance in Potato
Source: Plants (Basel). 2023 Feb 7;12(4):734. doi: 10.3390/plants12040734 (PMC9963855; doi:10.3390/plants12040734)
Supplement: Supplementary file 1 [file plants-12-00734-s001.zip › Supplementary File 4.pdf]

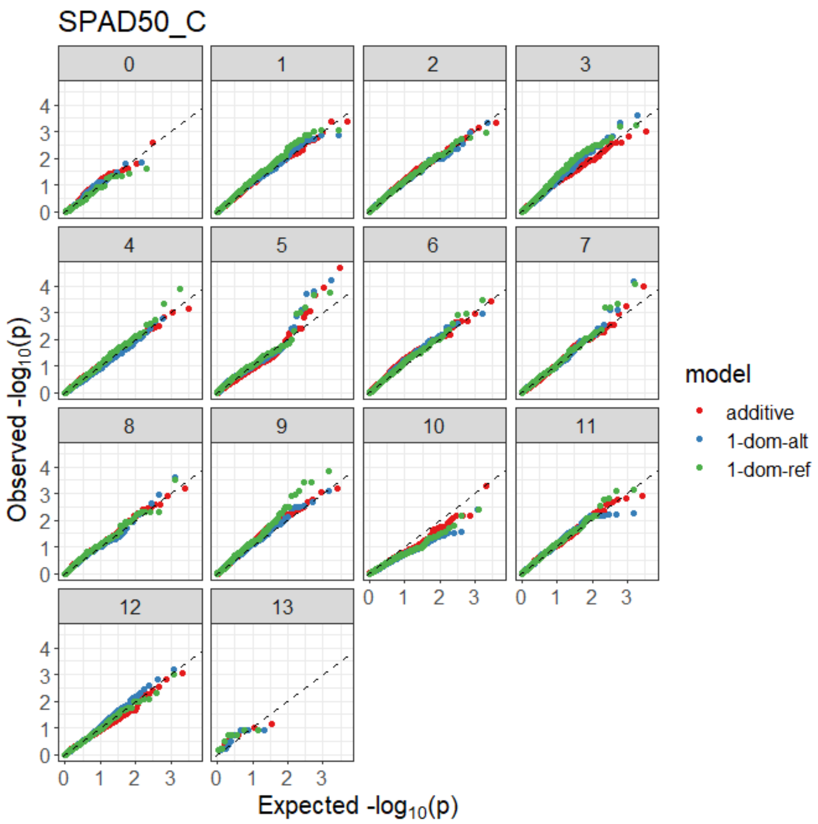
SPAD50_C
0
1
2
3
4
5
6
7
8
9
10
11
12
13
Observed -log10(p)
Expected -log10(p)
model
additive
1-dom-alt
1-dom-ref

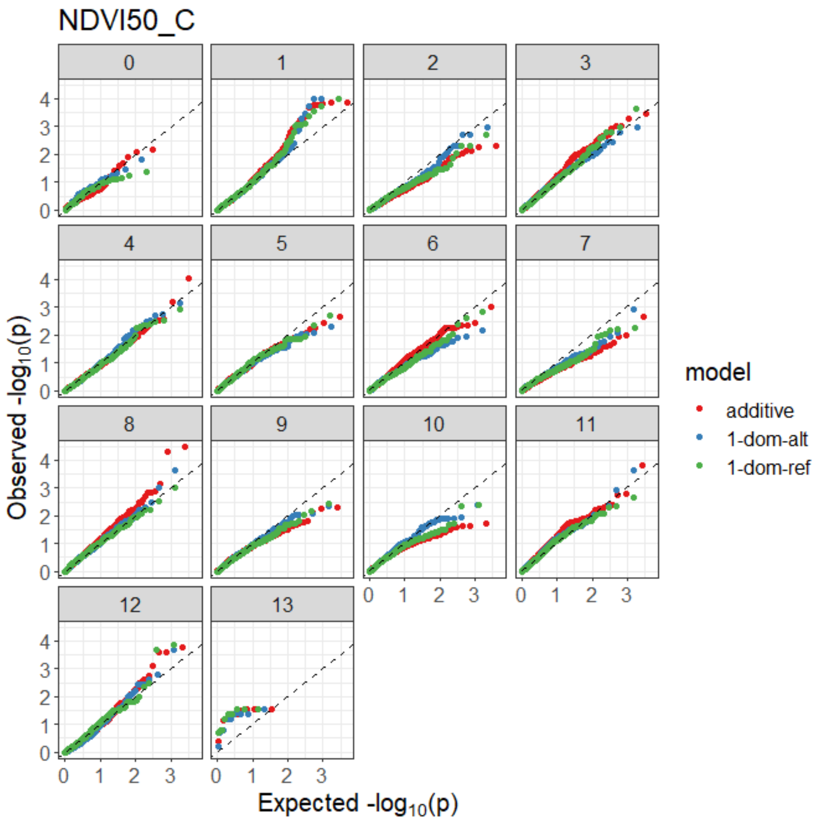
NDVI50_C
0
1
2
3
4
5
6
7
8
9
10
11
12
13
Observed -log10(p)
Expected -log10(p)
model
additive
1-dom-alt
1-dom-ref

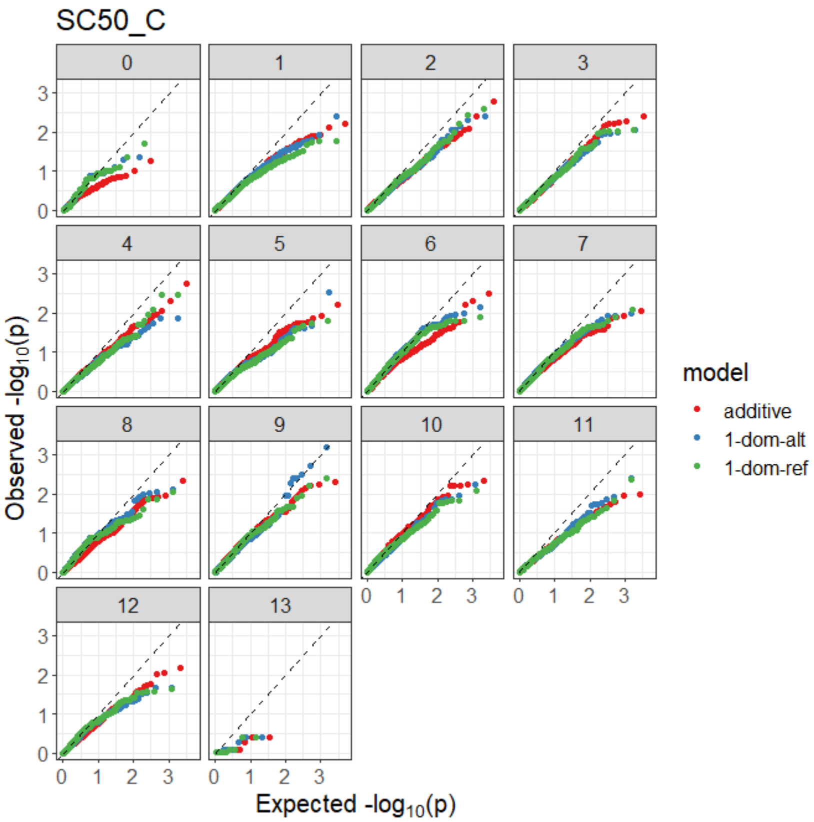
SC50_C
Observed -log10(p)
Expected -log10(p)
model
additive
1-dom-alt
1-dom-ref

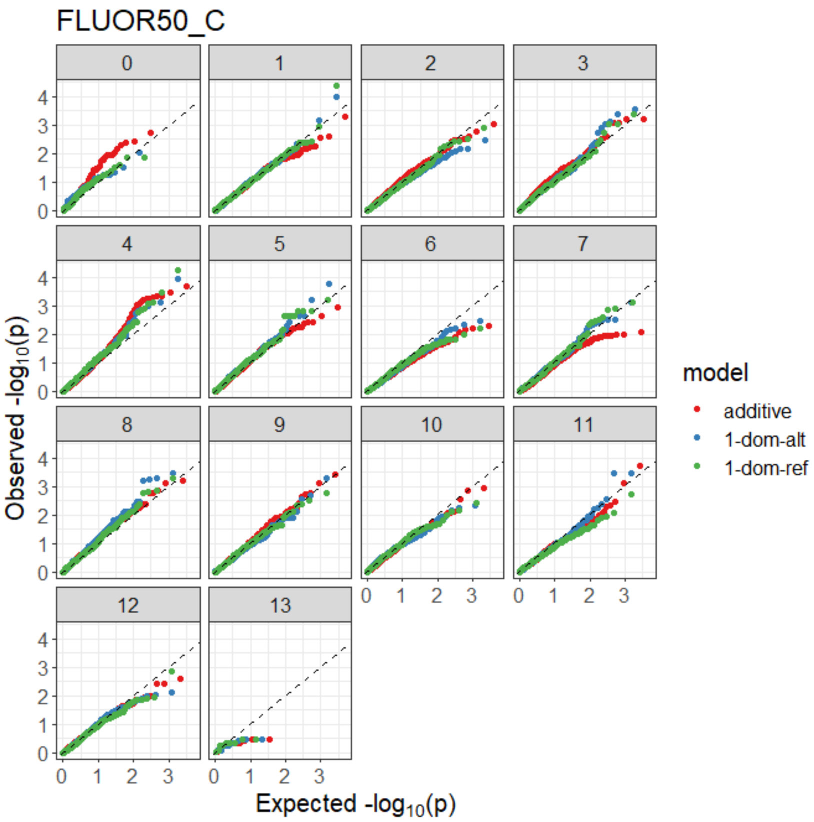
FLUOR50_C
Observed -log10(p)
Expected -log10(p)
model
additive
1-dom-alt
1-dom-ref

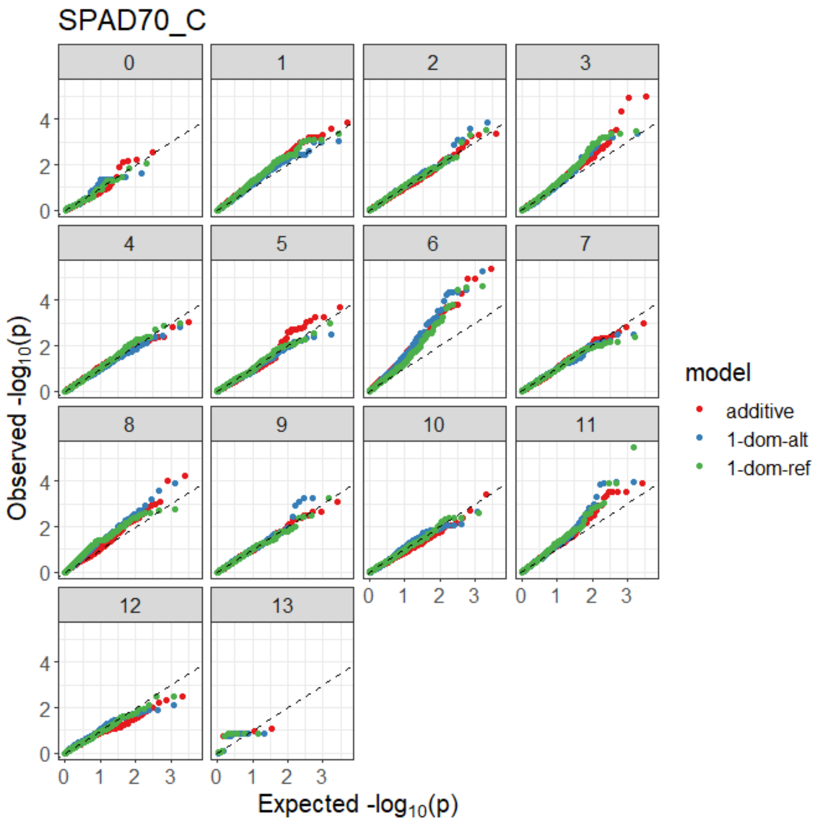

SPAD70_C
0
1
2
3
4
5
6
7
8
9
10
11
12
13
Observed $-\log_{10}(p)$
Expected $-\log_{10}(p)$
model
additive
1-dom-alt
1-dom-ref

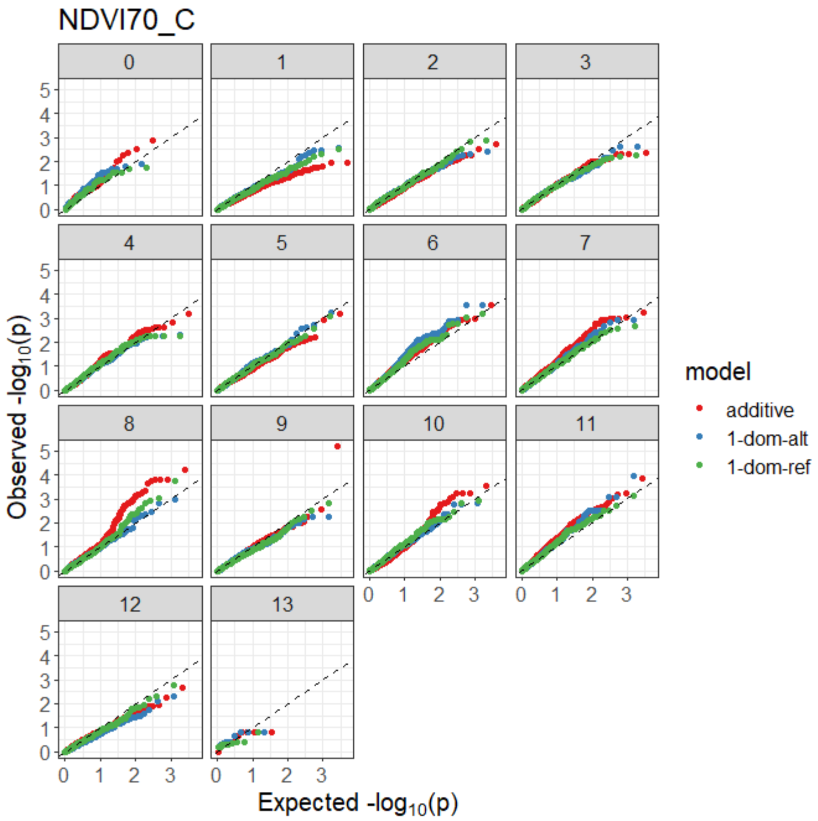

NDVI70_C
0
1
2
3
4
5
6
7
8
9
10
11
12
13
Observed $-\log_{10}(p)$
Expected $-\log_{10}(p)$
model
additive
1-dom-alt
1-dom-ref

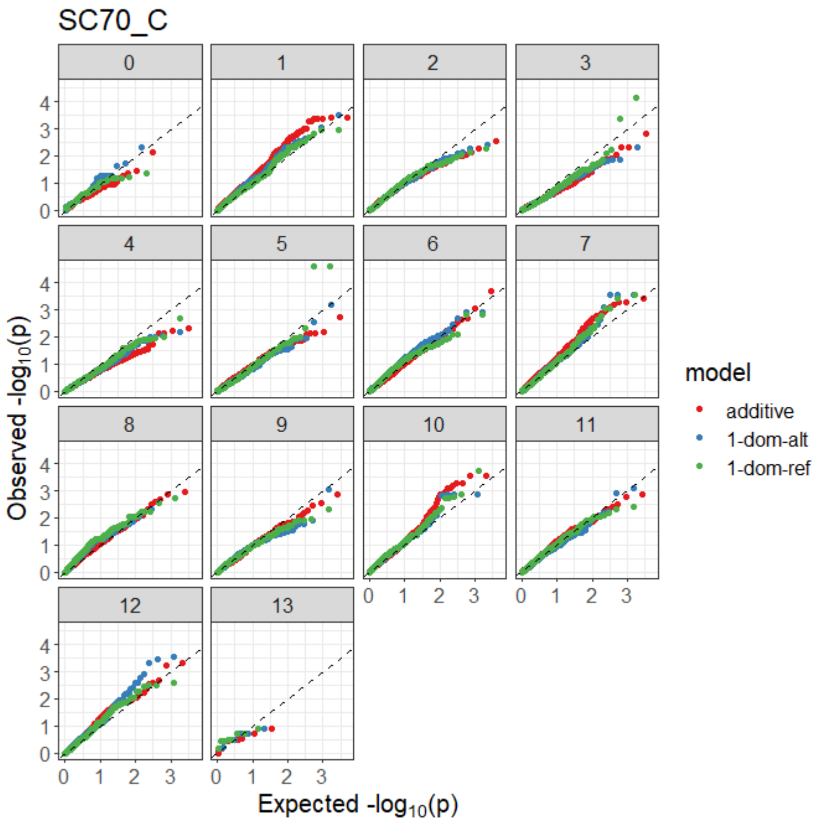
SC70_C
Observed -log10(p)
Expected -log10(p)
model
additive
1-dom-alt
1-dom-ref

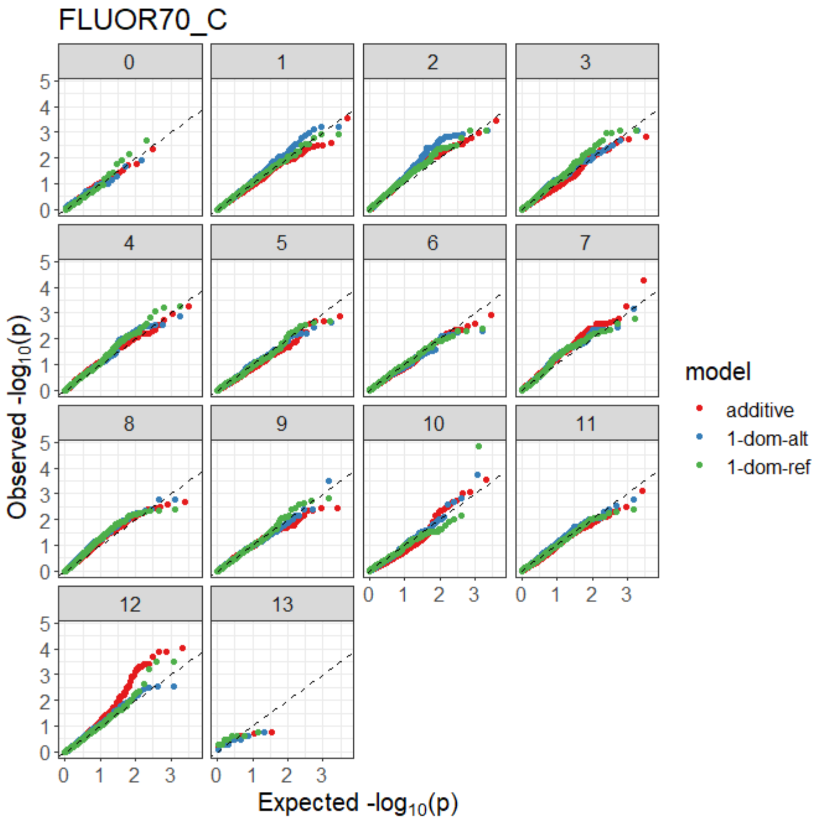
FLUOR70_C
Observed -log10(p)
Expected -log10(p)
model
additive
1-dom-alt
1-dom-ref

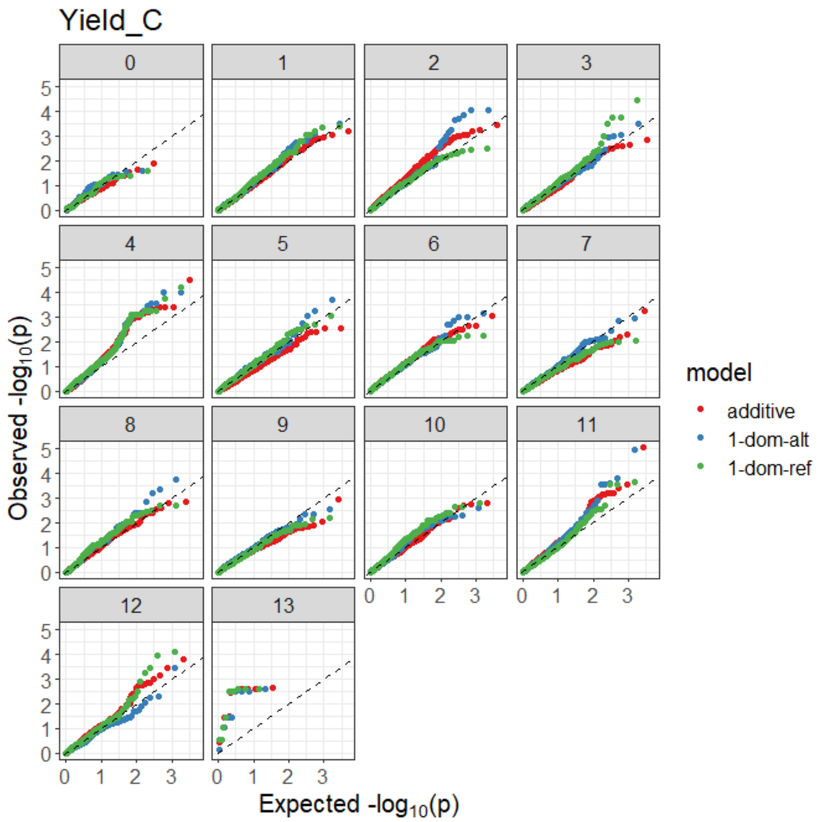
Yield_C
0
1
2
3
4
5
6
7
8
9
10
11
12
13
Observed -log10(p)
Expected -log10(p)
model
additive
1-dom-alt
1-dom-ref

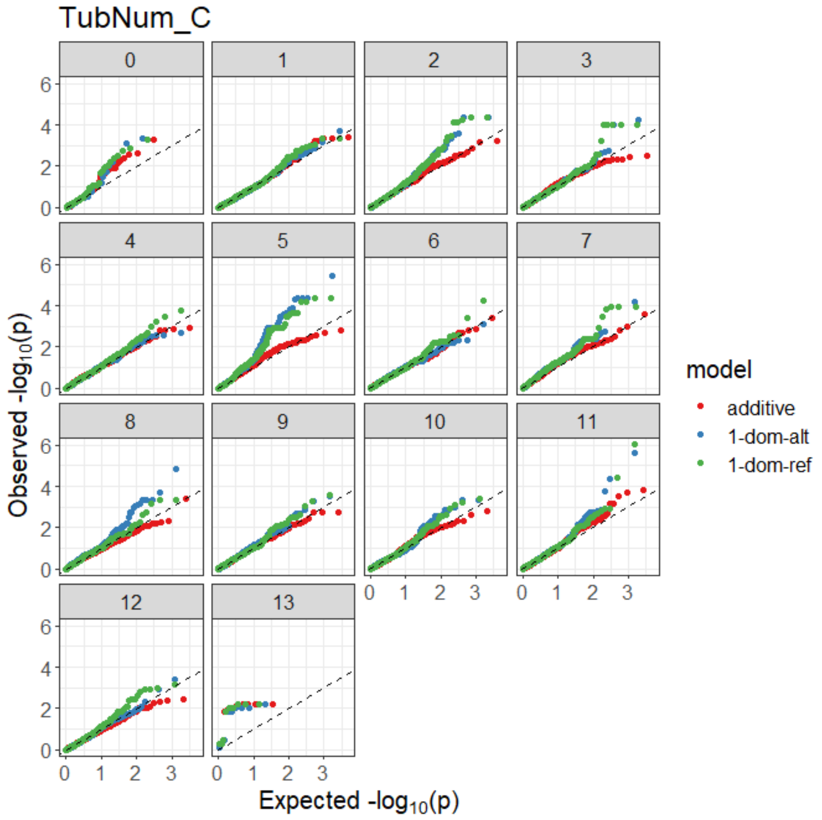
TubNum_C
0
1
2
3
4
5
6
7
8
9
10
11
12
13
Observed -log10(p)
Expected -log10(p)
model
additive
1-dom-alt
1-dom-ref

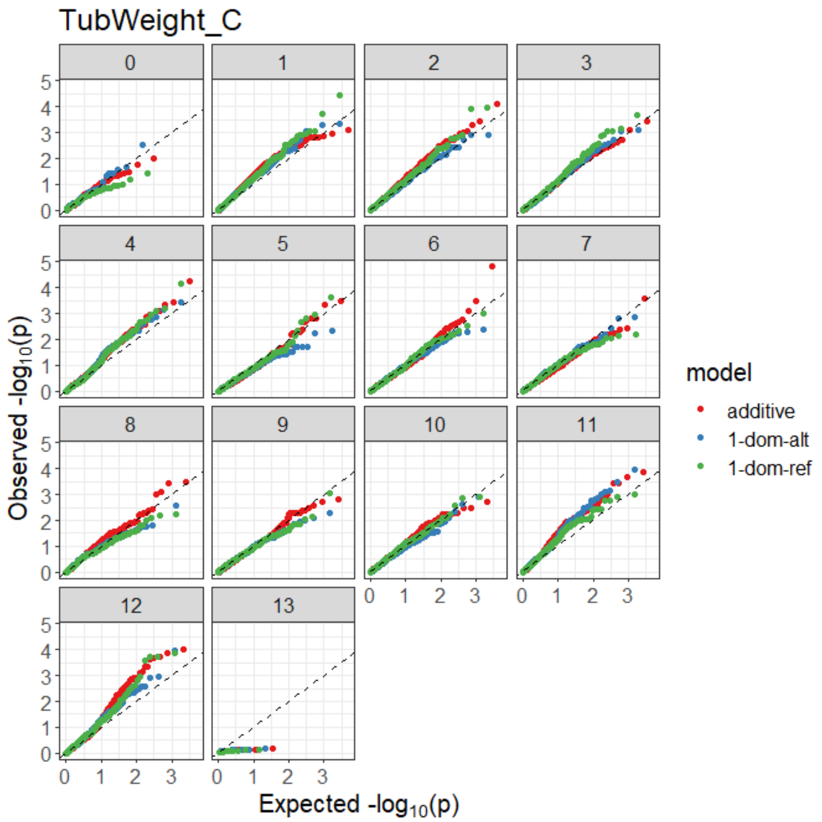

TubWeight_C
0
1
2
3
4
5
6
7
8
9
10
11
12
13
Observed -log10(p)
Expected -log10(p)
model
additive
1-dom-alt
1-dom-ref

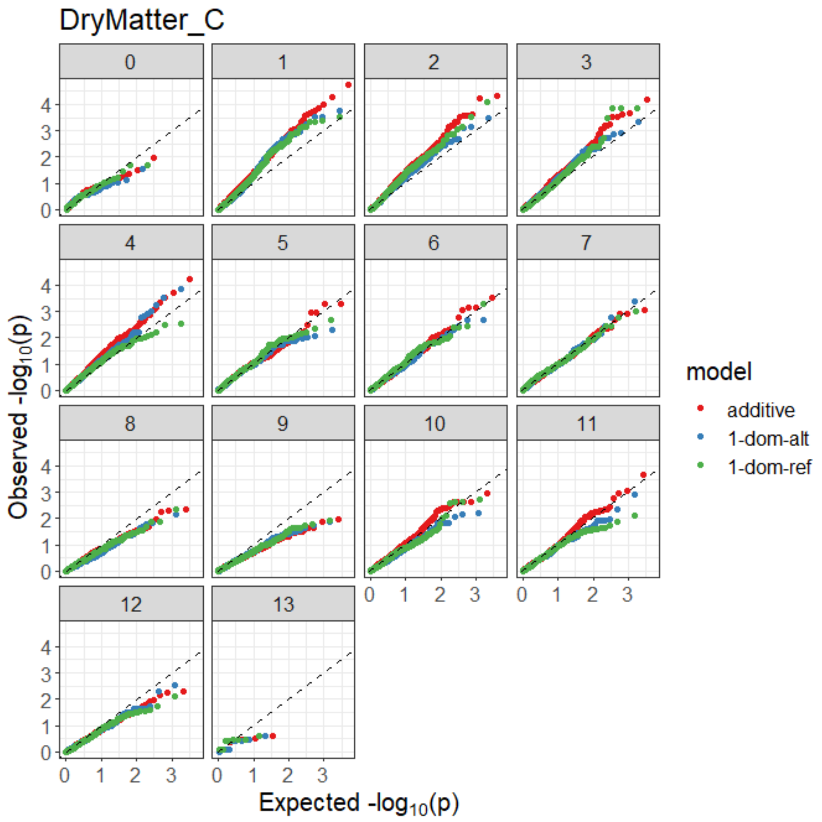

DryMatter_C
0
1
2
3
4
5
6
7
8
9
10
11
12
13
Observed -log10(p)
Expected -log10(p)
model
additive
1-dom-alt
1-dom-ref

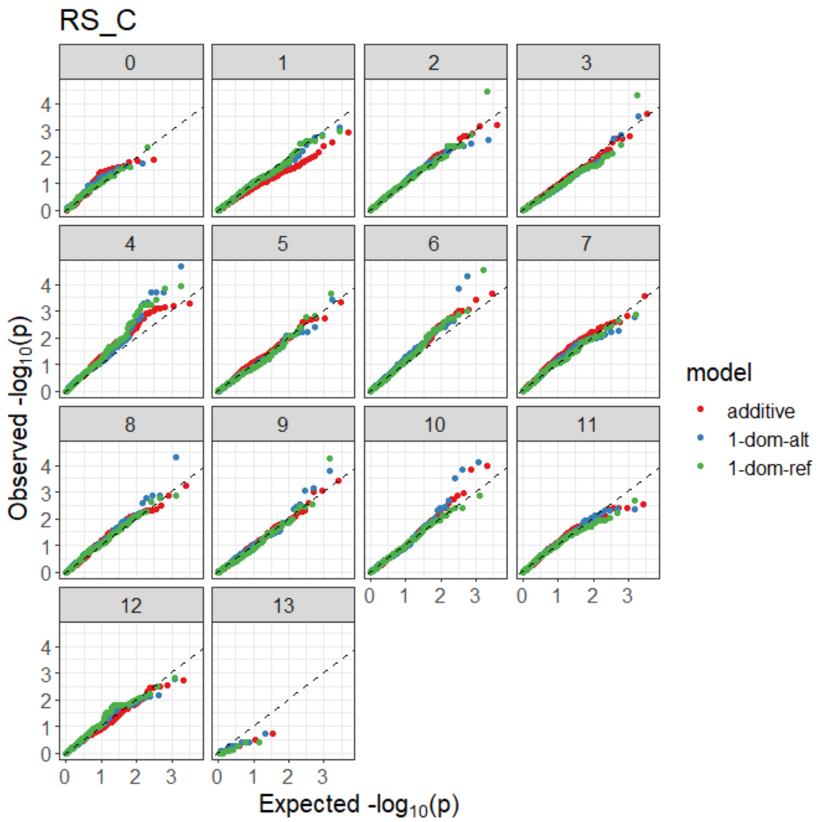
RS_C
0
1
2
3
4
5
6
7
8
9
10
11
12
13
Observed -log10(p)
Expected -log10(p)
model
additive
1-dom-alt
1-dom-ref

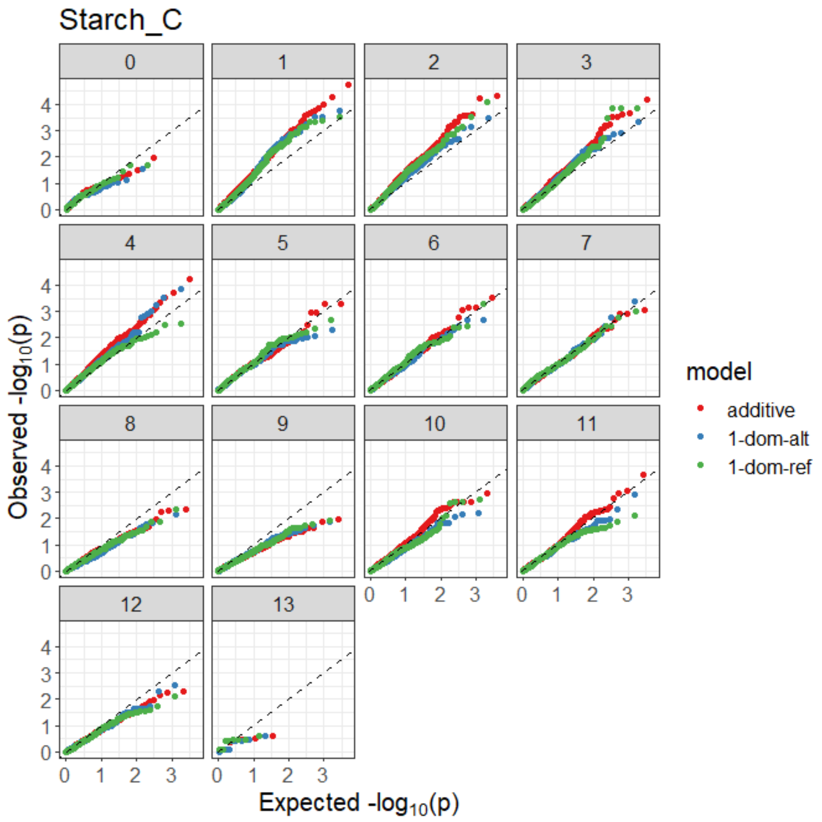
Starch_C
0
1
2
3
4
5
6
7
8
9
10
11
12
13
Observed -log10(p)
Expected -log10(p)
model
additive
1-dom-alt
1-dom-ref

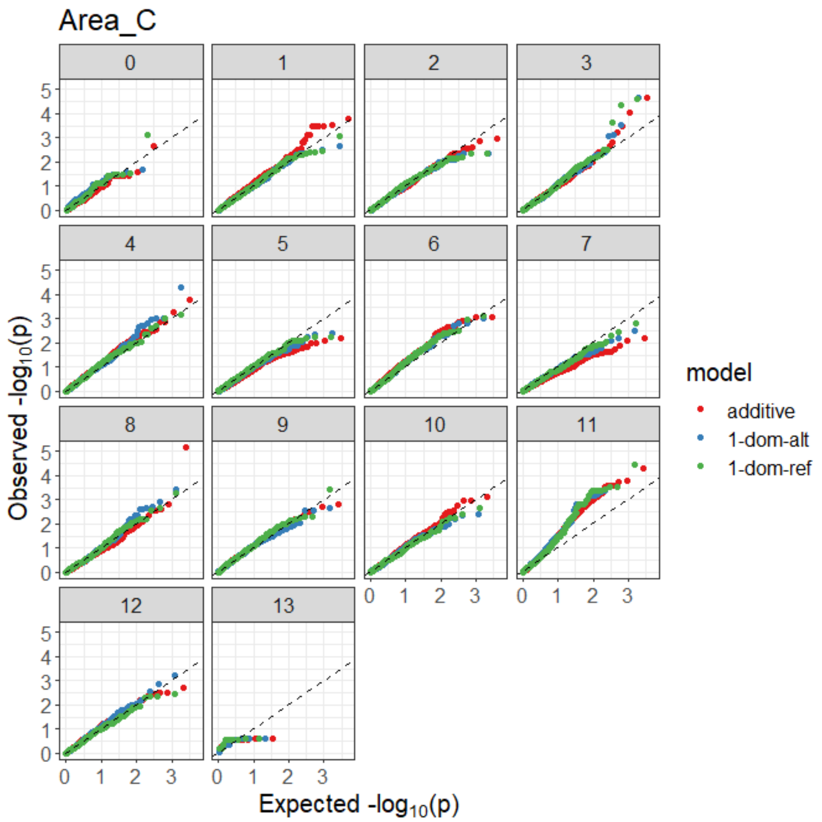
Area_C
0
1
2
3
4
5
6
7
8
9
10
11
12
13
Observed -log10(p)
Expected -log10(p)
model
additive
1-dom-alt
1-dom-ref

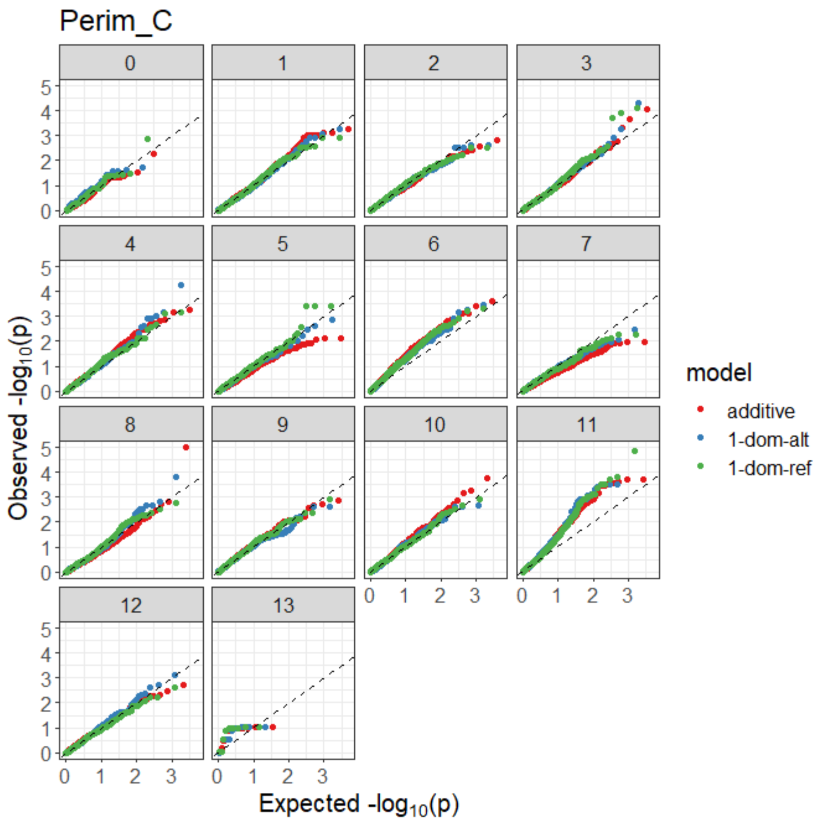
Perim_C
0
1
2
3
4
5
6
7
8
9
10
11
12
13
Observed -log10(p)
Expected -log10(p)
model
additive
1-dom-alt
1-dom-ref

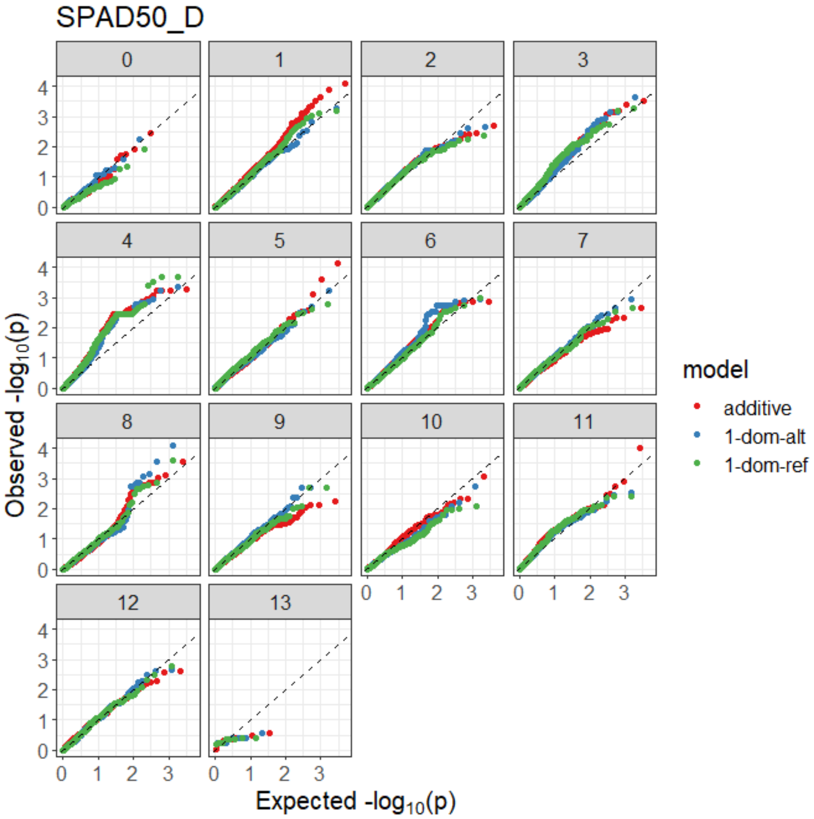
SPAD50_D
0
1
2
3
4
5
6
7
8
9
10
11
12
13
Observed -log10(p)
Expected -log10(p)
model
additive
1-dom-alt
1-dom-ref

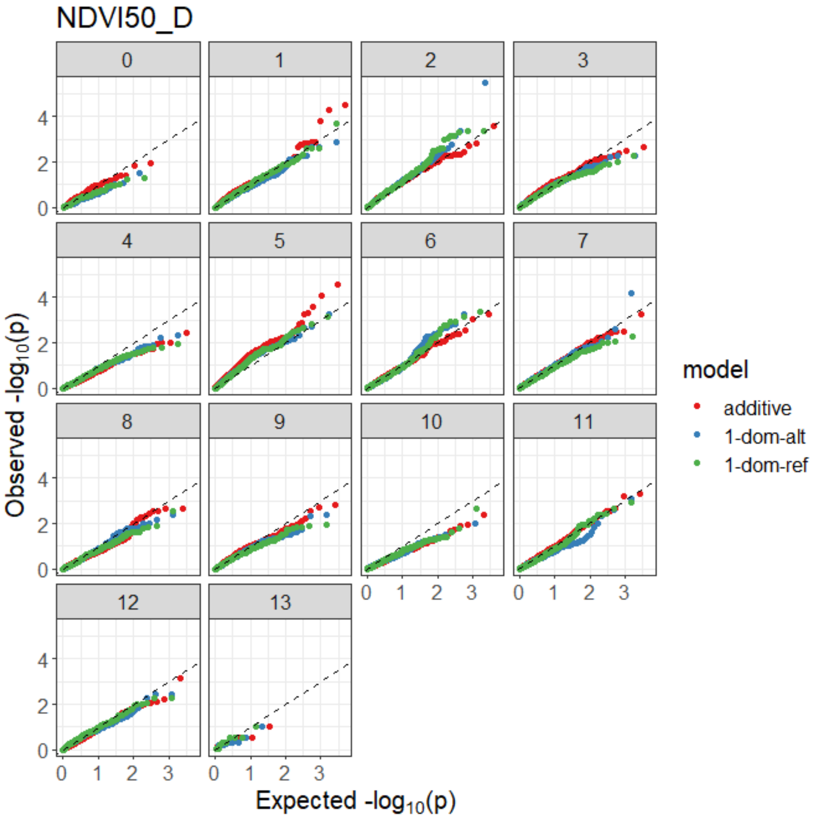
NDVI50_D
0
1
2
3
4
5
6
7
8
9
10
11
12
13
Observed -log10(p)
Expected -log10(p)
model
additive
1-dom-alt
1-dom-ref

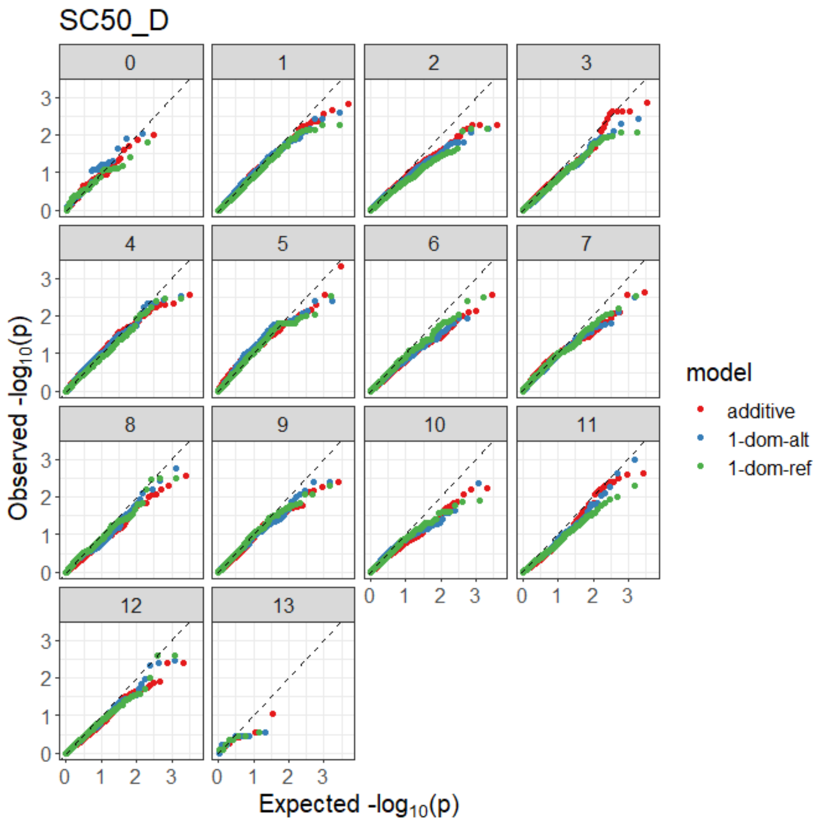
SC50_D
0
1
2
3
4
5
6
7
8
9
10
11
12
13
Observed -log10(p)
Expected -log10(p)
model
additive
1-dom-alt
1-dom-ref

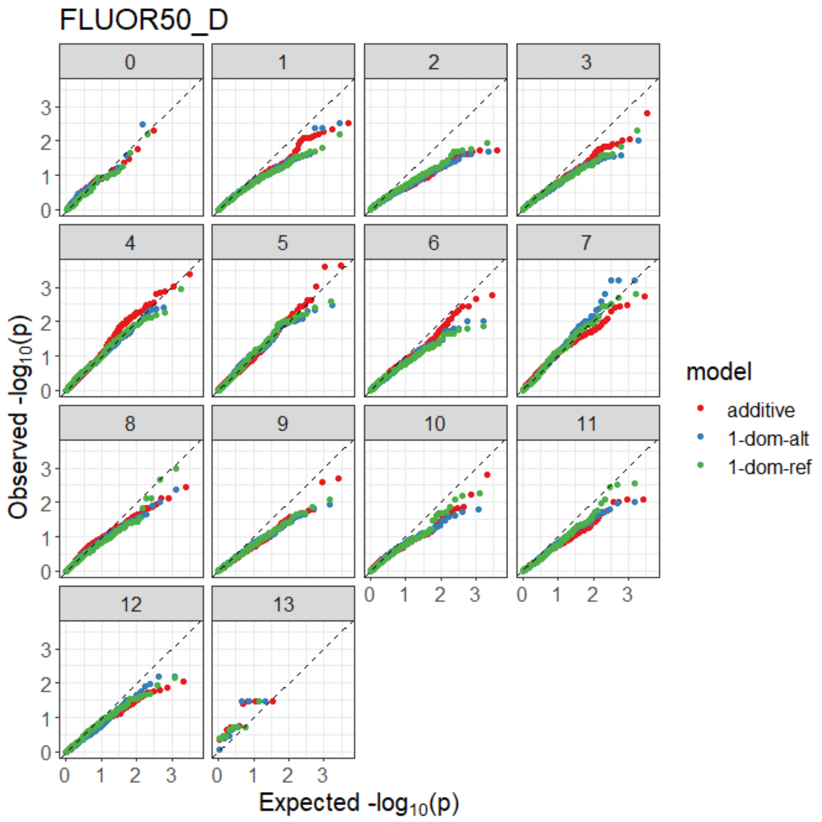
FLUOR50_D
0
1
2
3
4
5
6
7
8
9
10
11
12
13
Observed -log10(p)
Expected -log10(p)
model
additive
1-dom-alt
1-dom-ref

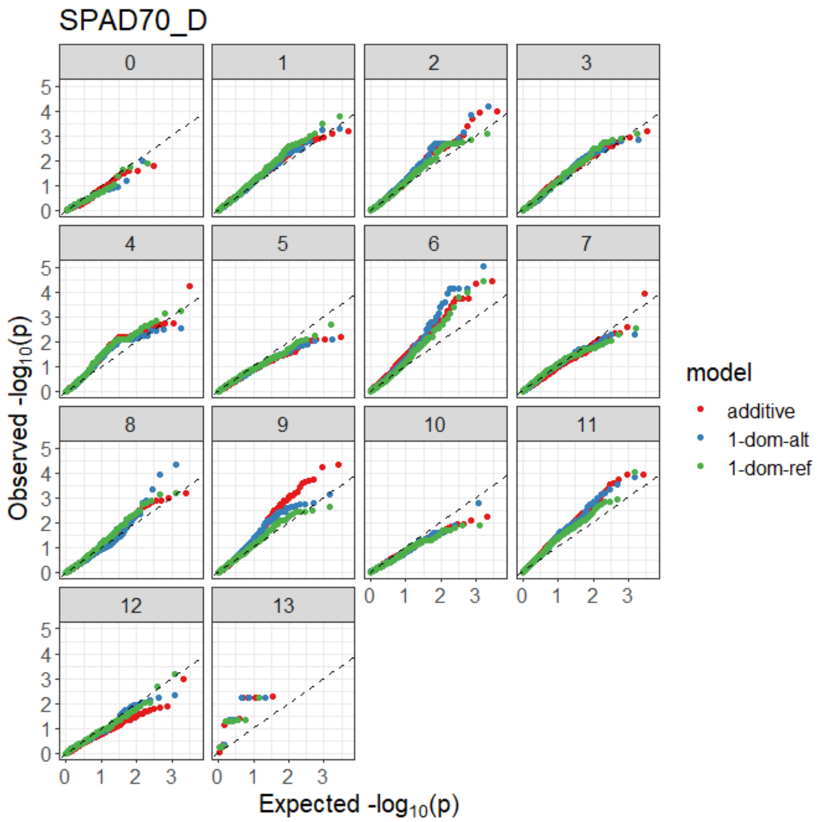
SPAD70_D
Observed -log10(p)
Expected -log10(p)
model
additive
1-dom-alt
1-dom-ref

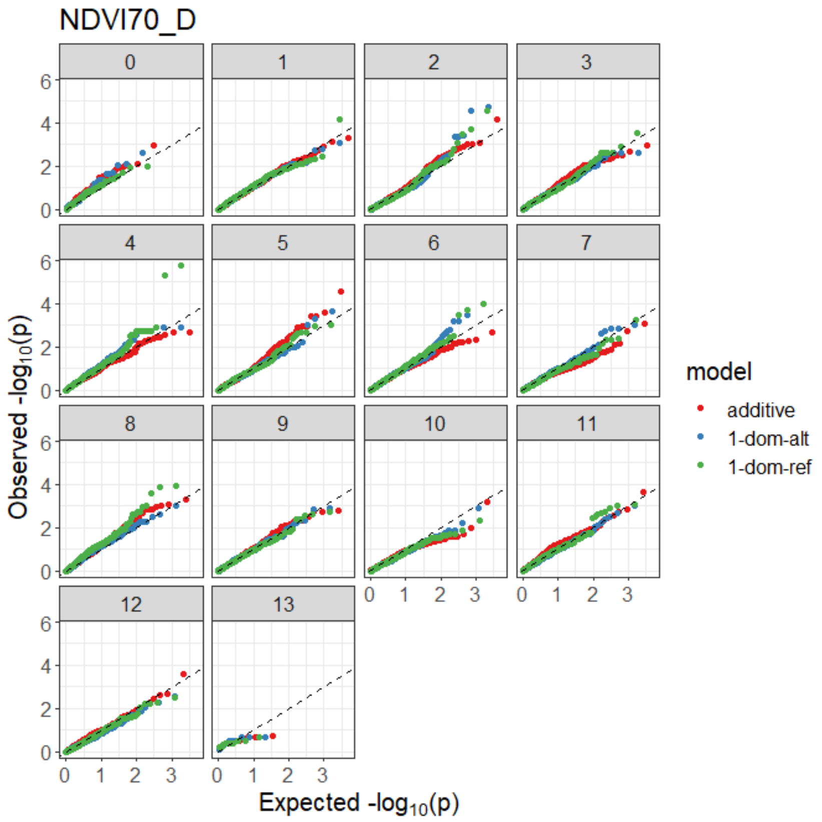
NDVI70_D
Observed -log10(p)
Expected -log10(p)
model
additive
1-dom-alt
1-dom-ref

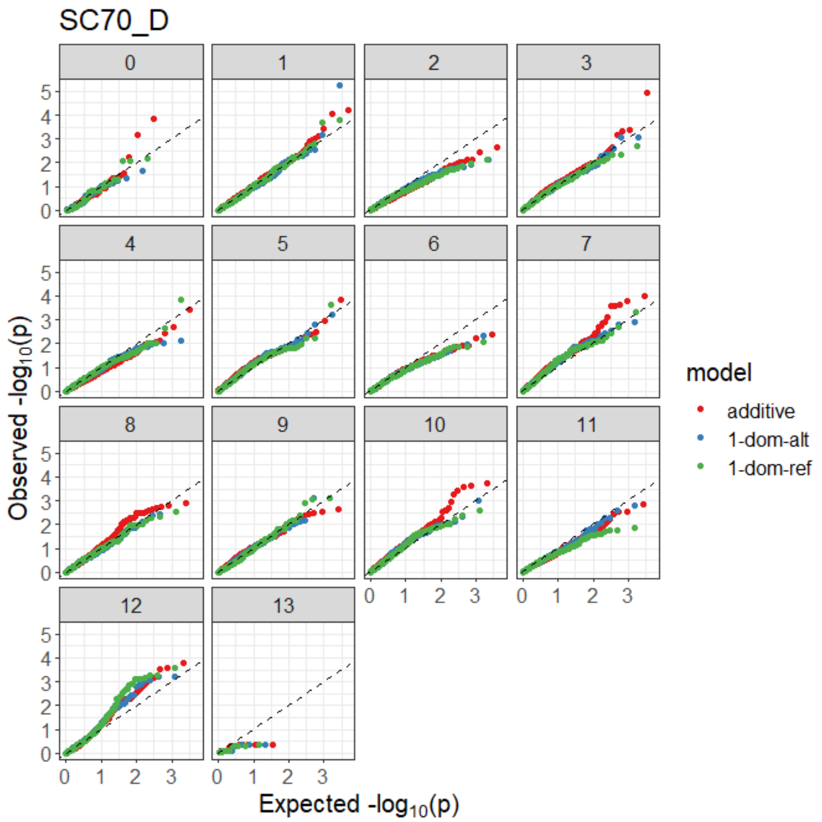
SC70_D
0
1
2
3
4
5
6
7
8
9
10
11
12
13
Observed -log10(p)
Expected -log10(p)
model
additive
1-dom-alt
1-dom-ref

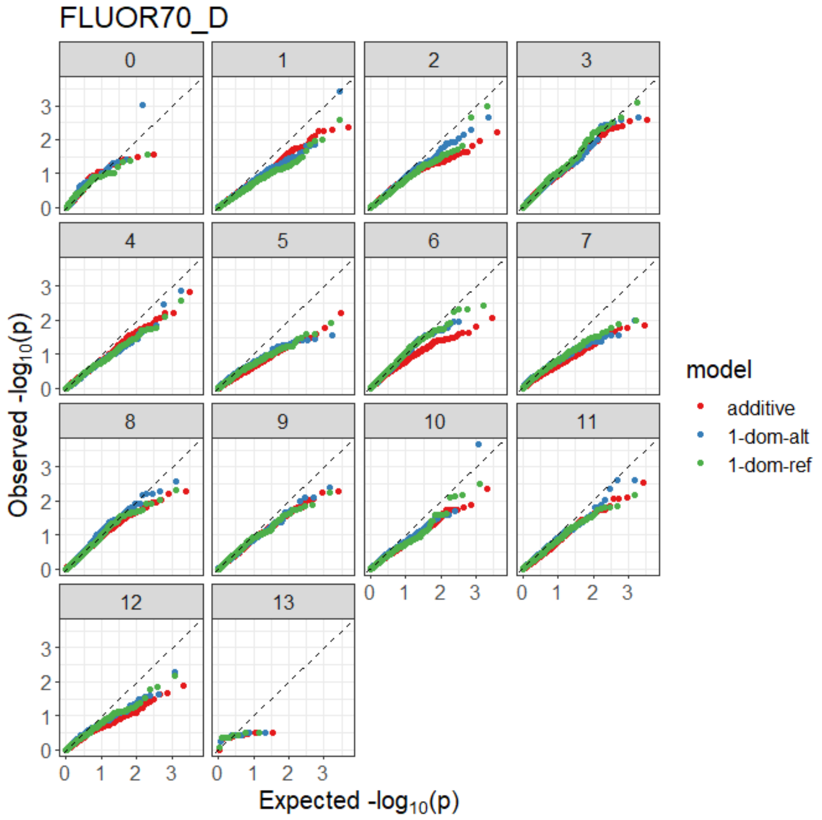
FLUOR70_D
0
1
2
3
4
5
6
7
8
9
10
11
12
13
Observed -log10(p)
Expected -log10(p)
model
additive
1-dom-alt
1-dom-ref

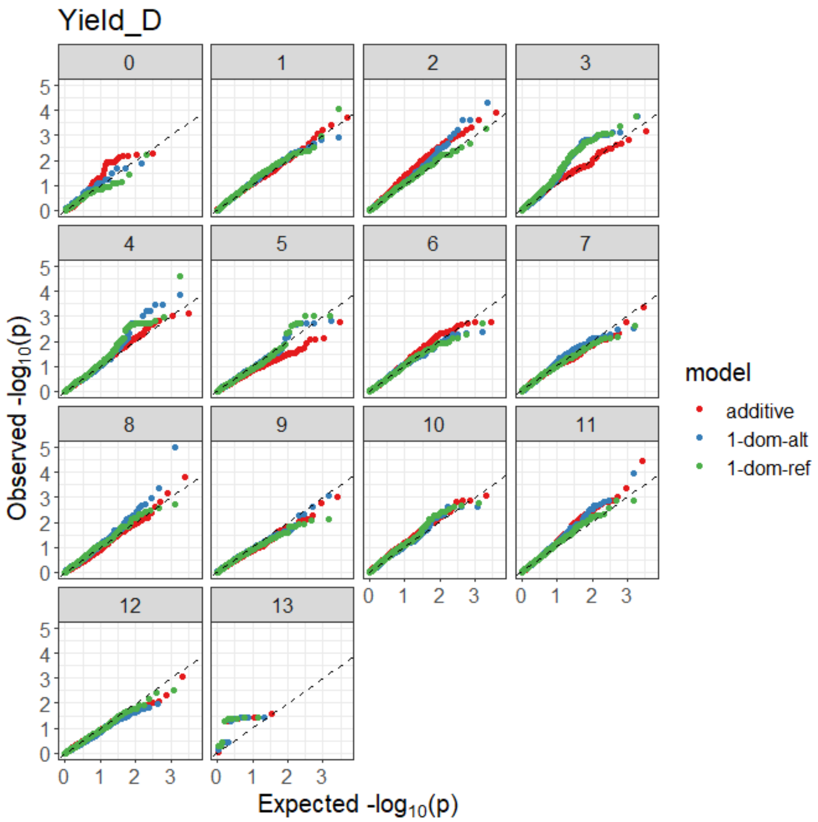
Yield_D
0
1
2
3
4
5
6
7
8
9
10
11
12
13
Observed $-log_{10}(p)$
Expected $-log_{10}(p)$
model
additive
1-dom-alt
1-dom-ref

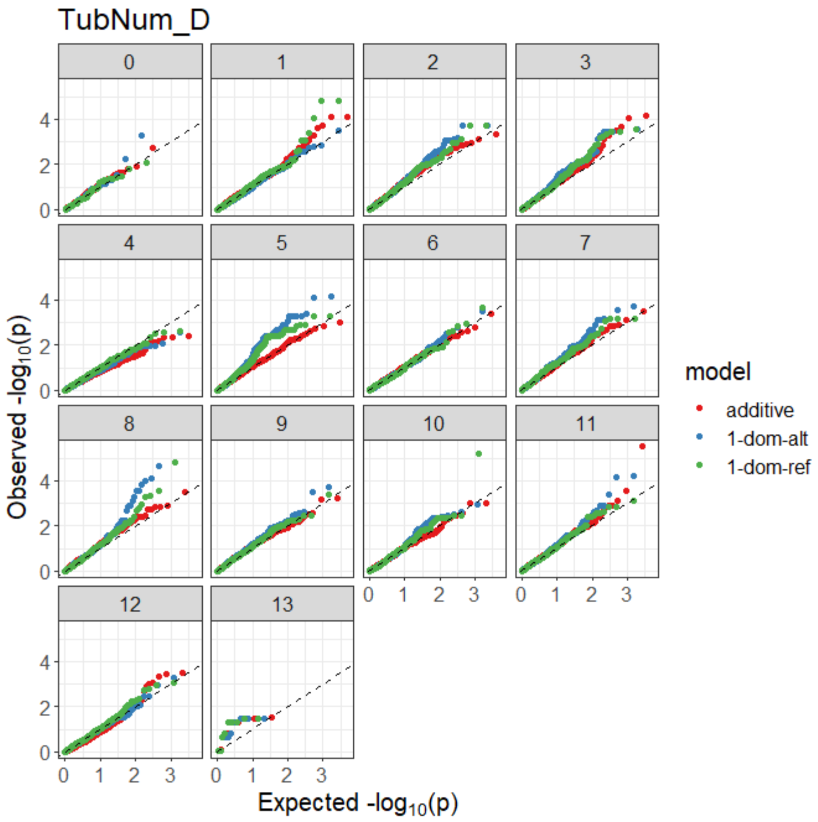
TubNum_D
0
1
2
3
4
5
6
7
8
9
10
11
12
13
Observed $-log_{10}(p)$
Expected $-log_{10}(p)$
model
additive
1-dom-alt
1-dom-ref

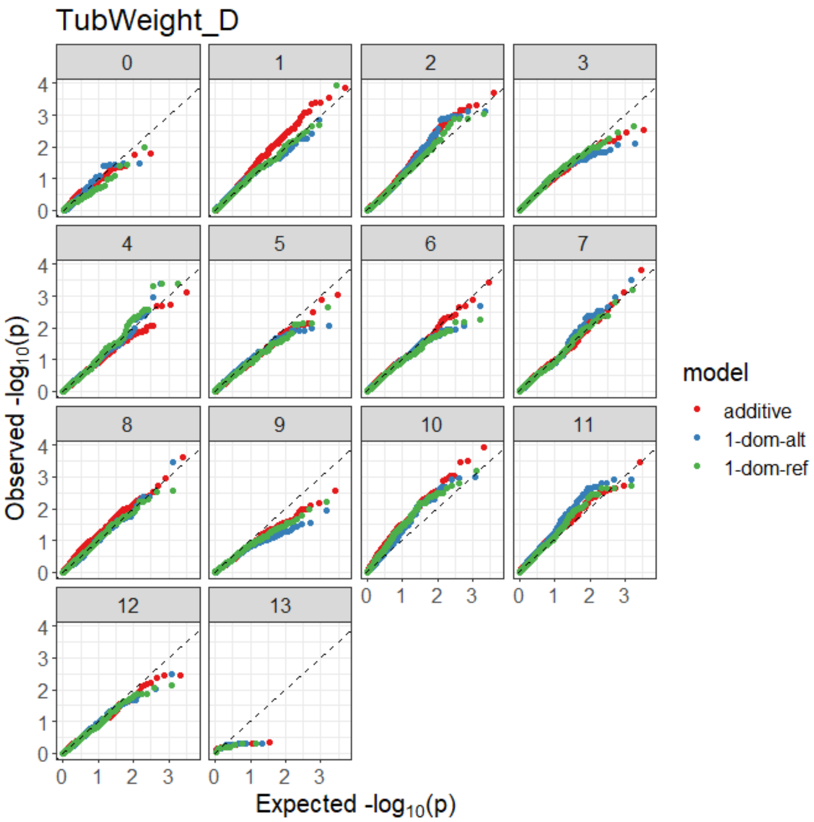
TubWeight_D
0
1
2
3
4
5
6
7
8
9
10
11
12
13
Observed -log10(p)
Expected -log10(p)
model
additive
1-dom-alt
1-dom-ref

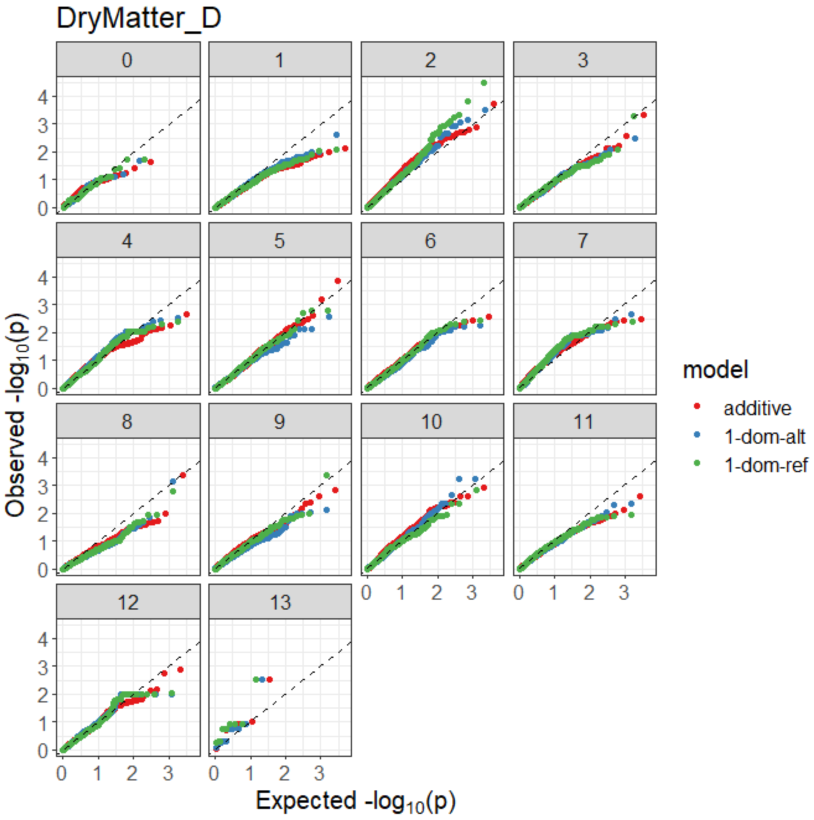
DryMatter_D
0
1
2
3
4
5
6
7
8
9
10
11
12
13
Observed -log10(p)
Expected -log10(p)
model
additive
1-dom-alt
1-dom-ref

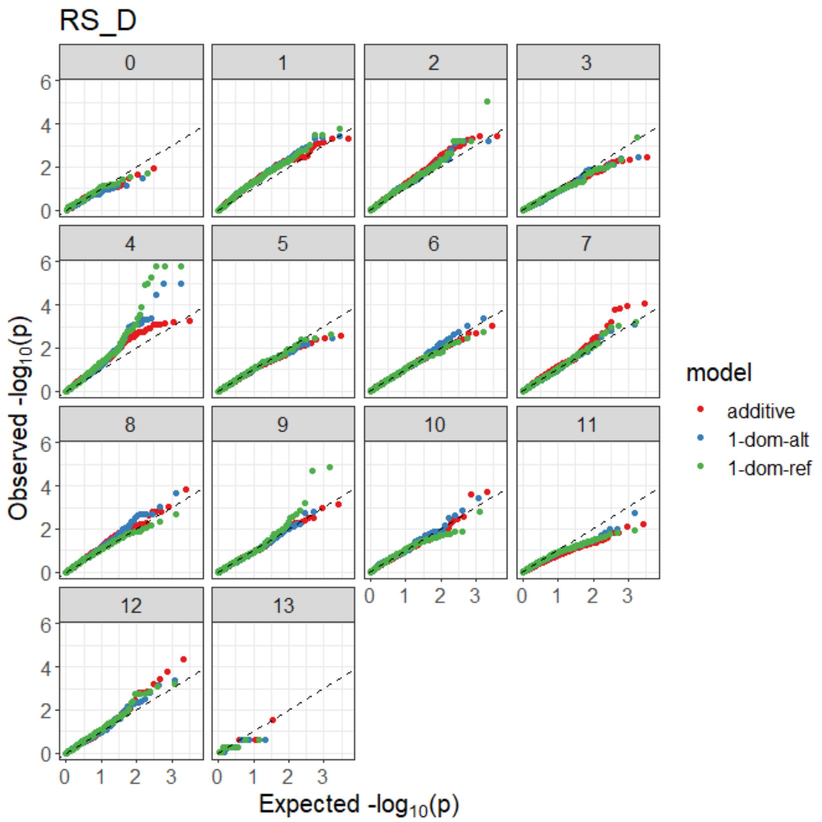
RS_D
0
1
2
3
4
5
6
7
8
9
10
11
12
13
Observed -log10(p)
Expected -log10(p)
model
additive
1-dom-alt
1-dom-ref

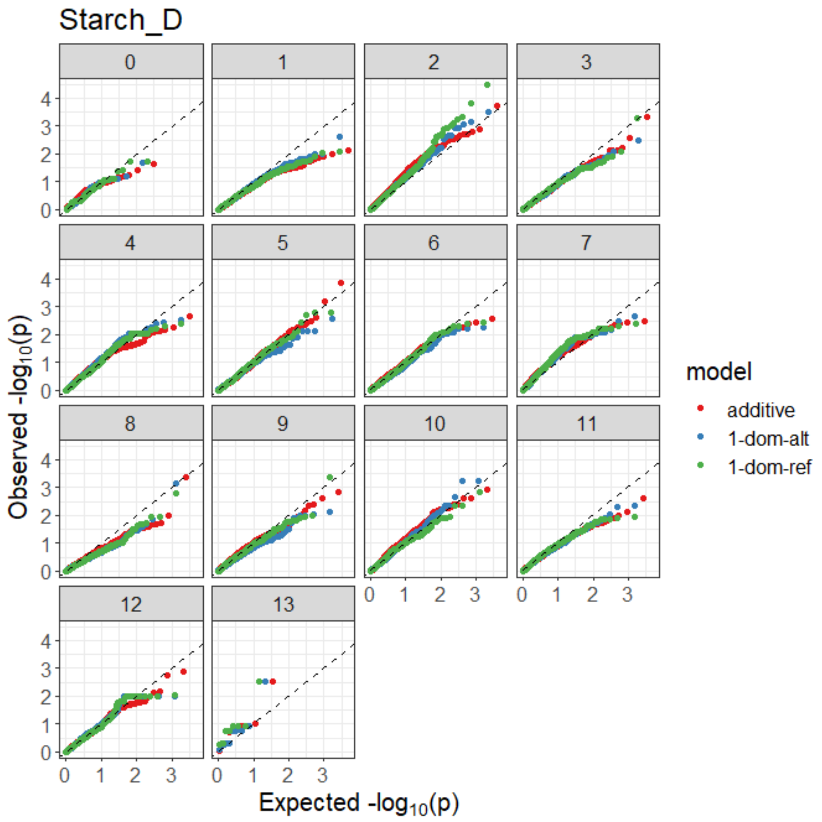
Starch_D
0
1
2
3
4
5
6
7
8
9
10
11
12
13
Observed -log10(p)
Expected -log10(p)
model
additive
1-dom-alt
1-dom-ref

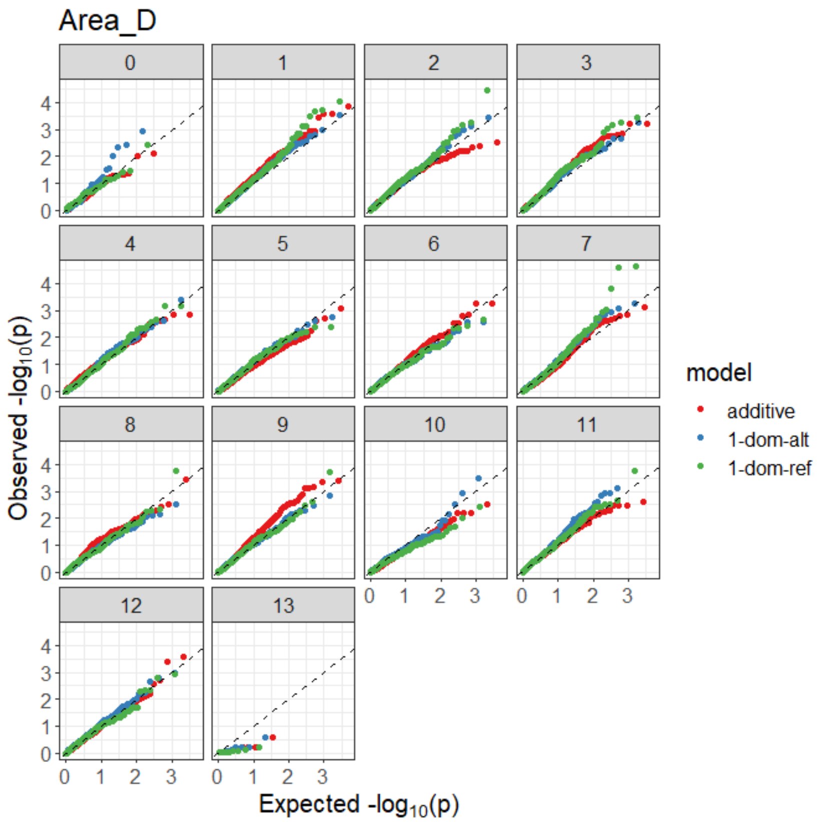

Area_D
model
additive
1-dom-alt
1-dom-ref
Observed -log10(p)
Expected -log10(p)

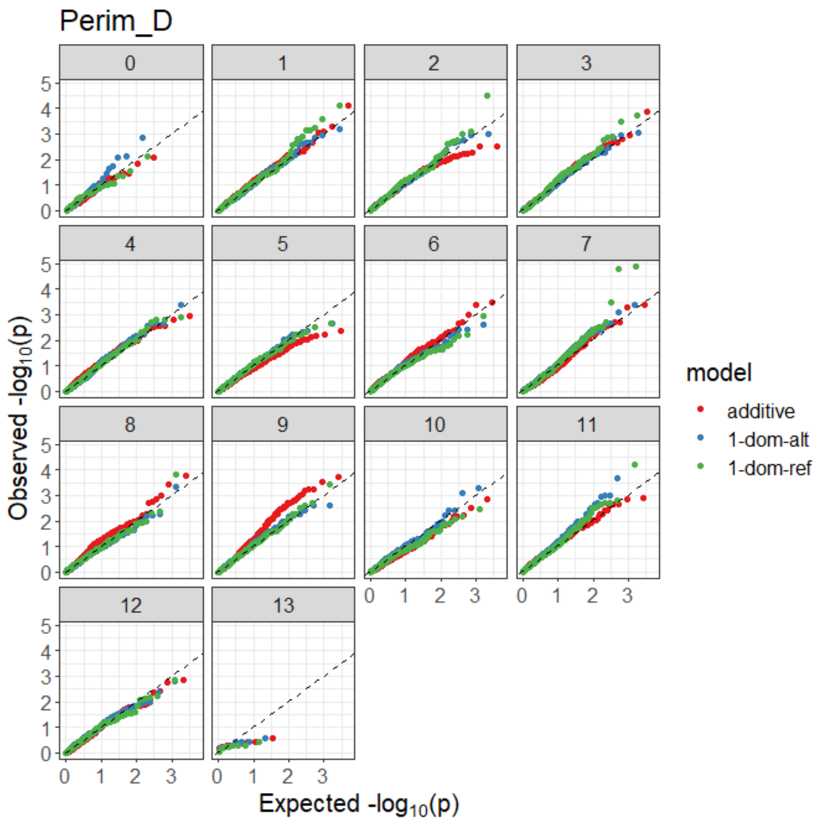

Perim_D
model
additive
1-dom-alt
1-dom-ref
Observed -log10(p)
Expected -log10(p)

Supplementary Figure S3. Q-Q plots for all traits evaluated under control and drought stress conditions in 144 potato varieties. 1 to 12 refers to each of the 12 potato chromosomes, 0 are control markers that are not associated with any chromosome and 13 refers to the chloroplast.
