# Supplementary figures and images for: Genome-Wide Association Study of Agronomic and Physiological Traits Related to Drought Tolerance in Potato
Source: Plants (Basel). 2023 Feb 7;12(4):734. doi: 10.3390/plants12040734 (PMC9963855; doi:10.3390/plants12040734)

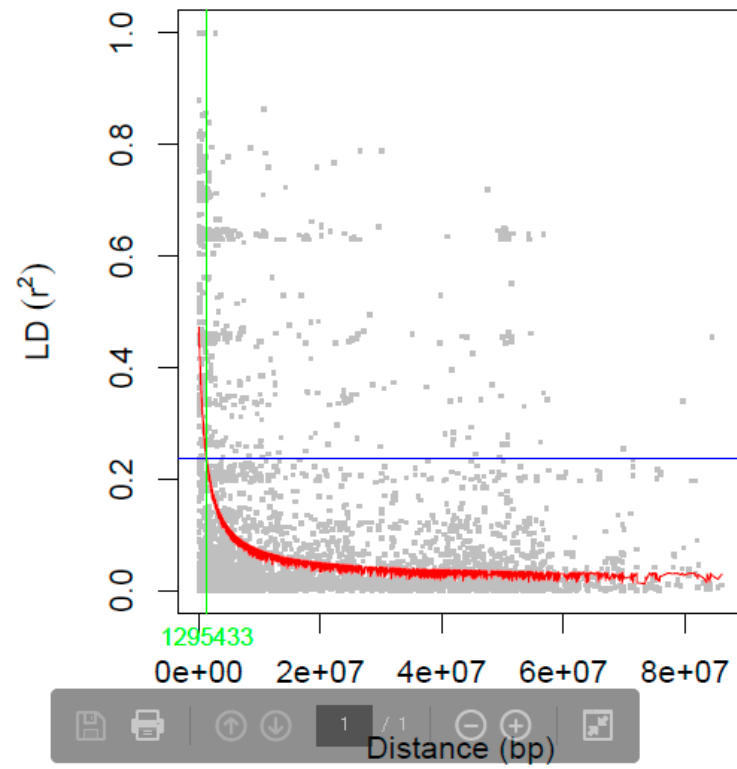

**Supplementary Figure S2.** Linkage disequilibrium (LD) decay plot between  $r^2$  and genetic distance.

Supplement: Supplementary file 1 [file plants-12-00734-s001.zip › Supplementary File 3.pdf]
